# A rare case of complete penoscrotal transposition with hypospadias in a newborn

**DOI:** 10.4274/tjod.88262

**Published:** 2017-03-15

**Authors:** Fatma Beyazıt, Eren Pek, Hakan Aylanç

**Affiliations:** 1 Çanakkale 18 Mart University Faculty of Medicine, Department of Obstetrics and Gynecology, Çanakkale, Turkey; 2 Çanakkale 18 Mart University Faculty of Medicine, Department of Pediatrics, Çanakkale, Turkey

**Keywords:** newborn, penoscrotal transposition, hypospadias

## Dear Editor,

Penoscrotal transposition (PST) is an infrequent congenital external genital malformation in which the scrotum is located superior and anterior to the penis^(1)^. PST can be defined as either complete or incomplete according to the positional exchanges between the penis and scrotum and both forms of PST are generally linked with hypospadias. Incomplete transposition is the common form of this entity and the penis lies in the middle of the scrotum, but in complete transposition, the scrotum almost entirely covers the penis, which emerges from the perineum^(2)^. Both of these conditions are commonly reported to be linked with a wide variety of abnormalities and pathologies that affect distinct organ systems. In this case report, we present a complete PST in a patient with urinary tract abnormalities including hypospadias, polycystic renal disease, and malpositioned right kidney.

A gravida 2, para 1 woman aged 35 years was admitted to the emergency department of our institute with premature membrane rupture when she was 36 weeks’ pregnant. The patient had undergone one prior cesarean delivery. The ultrasound examination revealed severe oligohydramnios. No previous histories of genetic abnormalities, illicit drug use, cigarette or alcohol consumption were reported. During her pregnancy, perinatal evaluations with ultrasound were performed at 26+6 weeks of gestation, which demonstrated a single umbilical artery, bilateral pelvic kidney, megacystitis, and penile curvature extending to the anal sphincter. Unfortunately, we were not able to obtain prenatal ultrasound pictures or a genetic analysis report because of the nature of the emergency hospital admission of the patient. A final prenatal diagnosis of PST and severe hypospadias was made based on these perinatal evaluations.

**Figure 1:**
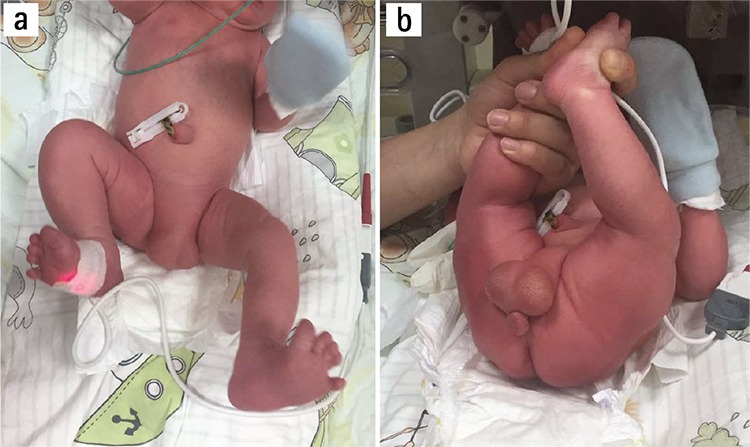
Complete penoscrotal transposition of a newborn a) Shows the scrotum without the penis b) Showing the penis under the scrotum

The patient underwent a cesarean section and gave birth to a male baby of 3070 grams with 8/9 Apgar score. Physical examination revealed a PST and severe hypospadias (Figure 1). Laboratory examinations were all reported to be normal. Ultrasonographic examination revealed a malpositioned right kidney (low lying) with multiple anechoic cysts of varying sizes. The newborn was transferred to the neonatal intensive care unit for further treatment.

PST is a very rare clinical situation in which the scrotum is located anterior and superior to the penis and a severe degree of PST, as in our case, with hypospadias and normal scrotum have been infrequently reported in medical literature^(1,3,4)^. During normal human maturation, scrotal swellings move inferomedially during the 9^th^-11^th^ week, and fuse in the midline caudal to the penis by the 12^th^ week of gestation^(4)^. The primary cause of this rare clinical disorder is a fusion defect or delay of urethral folds. Embryologically, PST is considered to result from abnormal genital tubercle development around the 6^th^-7^th^ week of gestation^(1)^.

The differential diagnosis for PST should include pseudohermaphroditism, micropenis, penile amputation in the intrauterine period, penoscrotal hypospadias, and agenesis of the penis accompanying a midline skin tag anterior to the anal region^(5)^. Moreover, a complete physical examination must be performed to detect abnormalities of the cardiovascular, central and peripheral nervous system, digestive system, urinary tract, and genital system because PST may present itself with a broad range of clinical manifestations that cause significant morbidity and mortality^(6)^. Surgery is the gold standard of PST management, which is usually preferred to be performed between 12-18 months. Although complete PST is rarely reported in the literature, considerable surgical skill is needed to reconstruct the penile anatomy.
